# Matrine Exerts a Strong Anti-Arthritic Effect on Type II Collagen-Induced Arthritis in Rats by Inhibiting Inflammatory Responses

**DOI:** 10.3390/ijms17091410

**Published:** 2016-08-26

**Authors:** Jiang Pu, Fan-Fu Fang, Xiu-Qing Li, Zhi-Heng Shu, Yi-Ping Jiang, Ting Han, Wei Peng, Cheng-Jian Zheng

**Affiliations:** 1 Administrative Office, Changhai Hospital, Second Military Medical University, 168 Changhai Road, Shanghai 200433, China; pujiang0408@163.com; 2 Department of Rehabilitation, Changhai Hospital of TCM, Second Military Medical University, Shanghai 200433, China; fangfanfu@126.com; 3 Department of Pharmacognosy, School of Pharmacy, Second Military Medical University, 325 Guohe Road, Shanghai 200433, China; liuxiuqing@126.com (X.-Q.L.); 13817760547@163.com (Z.-H.S.); msjyp@163.com (Y.-P.J.); than927@163.com (T.H.); 4 College of Pharmacy, Chengdu University of Traditional Chinese Medicine, Chengdu 610075, China

**Keywords:** matrine, *Sophora flavescens*, rheumatoid arthritis, anti-arthritic effect, type II collagen

## Abstract

To investigate anti-arthritic effects of matrine isolated from the roots of *S. flavescens* on type II collagen-induced arthritis (CIA) in rats and to explore its related potential mechanisms, CIA rats were established and administered with matrine (20, 40 or 80 mg/kg/days, for 30 days). Subsequently, blood was collected to determine serum levels of TNF-α, IL-1β, IL-6, IL-8, IL-17A, IL-10, MMP-2, MMP-3 and MMP-9, and hind paws and knee joints were collected for histopathological examination. Furthermore, indices of the thymus and spleen were determined, and synovial tissues were collected to determine the protein expressions of p-IκB, IκB, Cox-2 and iNOS. Our results indicated that matrine significantly suppressed inflammatory reactions and synovial tissue destruction. Matrine inhibited paw swelling, arthritis indices and weight loss in CIA rats. Additionally, matrine decreased the levels of TNF-α, IL-1β, IL-6, IL-8, IL-17A, MMP-2, MMP-3 and MMP-9. Matrine also down-regulated expressions of p-IκB, Cox-2, and iNOS but up-regulated IκB in synovial tissues in CIA rats. The results suggested matrine possesses an anti-arthritic effect in CIA rats via inhibiting the release of pro-inflammatory cytokines and proteins that promote the NF-κB pathway.

## 1. Introduction

Rheumatoid arthritis (RA), an intractable and highly prevalent autoimmune disease, is characterized by hyperplasia and inflammation of the synovial joints. RA can result in serious and irreversible destruction of articular cartilage and bone [1,2,3]. In addition, RA can also induce systemic organ damage in structures including the heart, lungs, kidneys and arteries [4,5]. Currently, the molecular mechanisms underlying the pathogenesis of RA are being extensively investigated, and it has become clear that RA is a disease mainly mediated by T cells, synovial fibroblasts, and dentritic cells [3]. However, there are few RA treatment strategies that are effective, reliable, and have low toxicity [6]. Clinically, glucocorticoids (GCs, such as dexamethasone), disease-modifying anti-rheumatic drugs (DMARDs, such as methotrexate) and non-steroidal anti-inflammatory drugs (NSAIDs, such as ibuprofen) are commonly used to relieve the inflammatory reactions of RA patients. However, prolonged use of these drugs could result in severe side effects, such as gastrointestinal ulcer, myelo-suppression, cardiac diseases, etc. [6,7].

Natural monomers or extracts that have been isolated from plants or herbs have been demonstrated to effectively treat various diseases with relatively low toxicity [8,9,10]. *Sophora flavescens* (Leguminosea) is a famous traditional Chinese medicine and has long been used for the treatment of jaundice, pruritus, dysentery, and various inflammatory disorders [11,12,13]. Matrine is one of the most important alkaloids identified in *S. flavescens*, and it performs a wide variety of pharmacological functions, including anti-inflammatory, anti-cancer/tumor, and anti-oxidant [14,15]. Our work was focused on supplying a set of candidate drugs that could serve as a resource for future screenings aimed at identifying clinically effective anti-RA drugs, and we have reported that some natural agents could potentially be used to treat RA [10,16,17]. As a part of our continuing efforts to identify novel plant-derived anti-RA candidate drugs, our preliminary experiments showed that matrine might confer a potential anti-arthritic effect. Therefore, the present study was designed to systemically evaluate the anti-arthritic effect of matrine in an experimental animal model of RA.

## 2. Results

### 2.1. Matrine Decreases Paw Swelling, Arthritis Indices and Weight Loss in Collagen-Induced Arthritis (CIA) Rats

**Figure 1:**
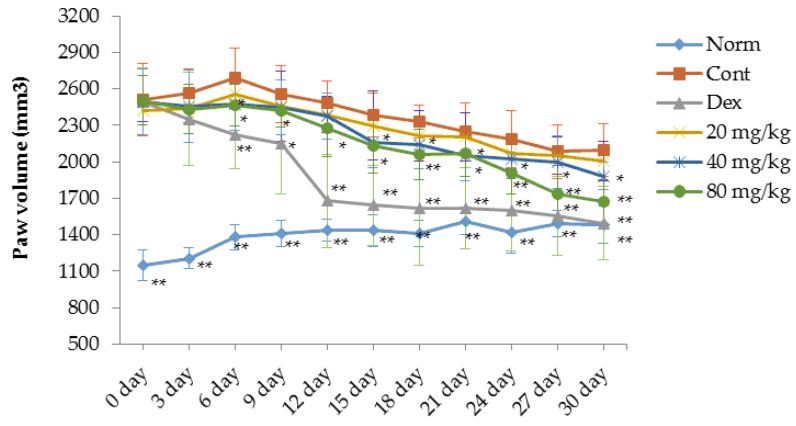
Effects of matrine on volume changes for the paw of CIA rats (mm^3^). Norm: normal rats; Cont: control rats; Dex: rats treated with dexamethasone. Data are presented as the mean ± SD (*n* = 10). * *p* < 0.05, ** *p* < 0.01, compared with Cont. Day 0 in the figure is day 21 after initial immunization.

**Figure 2:**
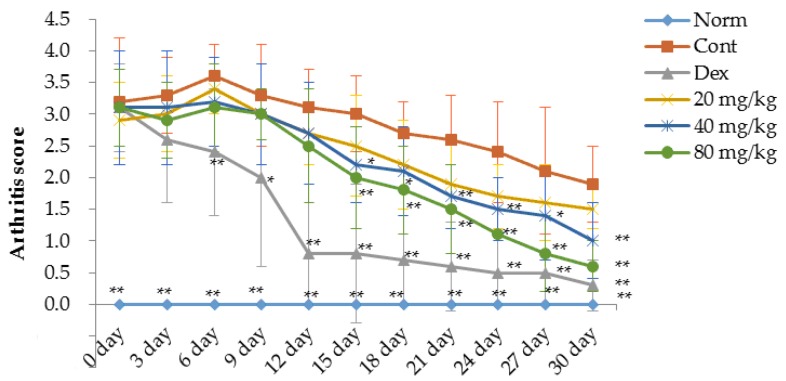
Effects of matrine on arthritis score changes for the paw of CIA rats. Norm: normal rats; Cont: control rats; Dex: rats treated with dexamethasone. Data are presented as the mean ± SD (*n* = 10). * *p* < 0.05, ** *p* < 0.01, compared with Cont. (control). Day 0 in the figure is day 21 after initial immunization.

**Figure 3:**
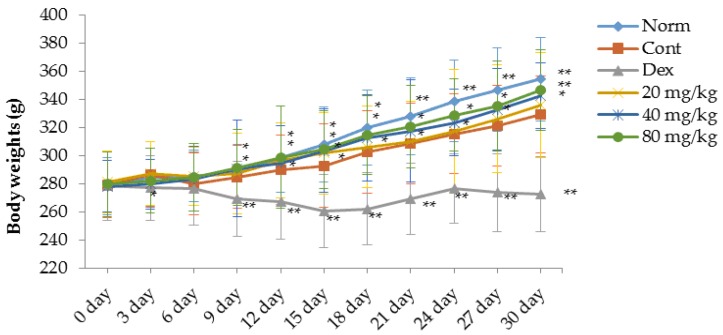
Effects of matrine on body weights changes of CIA rats. Norm: normal rats; Cont: control rats; Dex: rats treated with dexamethasone. Data are presented as the mean ± SD (*n* = 10). * *p* < 0.05, ** *p* < 0.01, compared with Cont. Day 0 in the figure is day 21 after initial immunization.

A type II collagen-induced arthritis (CIA) rat model was prepared to evaluate the therapeutic effects of matrine in rats with RA. As shown in Figure 1, Figure 2 and Figure 3, compared with the normal rats, significant RA symptoms were observed when the experimental rats were immunized with type II collagen (CII) twice, including paw swelling and erythema (*p* < 0.01), higher arthritis indices (*p* < 0.01) and obvious weight loss (*p* < 0.05). These symptoms significantly improved after treatment with dexamethasone. Our results indicate that starting on the 6th day after the initial dexamethasone administration (0.05 mg/kg), paw swelling was decreased in CIA rats compared with the control group (*p* < 0.01) (Figure 1). Additionally, the arthritis indices in the dexamethasone (0.05 mg/kg)-treated CIA rats were also significantly decreased compared with the control group (*p* < 0.01) (Figure 2). However, it was clear that the daily administration of dexamethasone (0.05 mg/kg) did not reverse the body weight loss observed in the CIA rats; instead, dexamethasone aggravated the weight loss (Figure 3). This effect might be associated with the inherent side effects of dexamethasone. Similar to the dexamethasone treatment, matrine significantly decreased paw swelling and arthritis indices when administered at doses of 40 (*p* < 0.05) and 80 mg/kg (*p* < 0.01), and matrine moderately decreased these symptoms when administered daily at a dose of 20 mg/kg (Figure 1 and Figure 2). Interestingly and importantly, the body weight loss observed in the CIA rats was reversed by daily treatment with matrine at doses of 20, 40 and 80 mg/kg (*p* < 0.05, *p* < 0.01, and *p* < 0.01, respectively) (Figure 3).

### 2.2. Matrine Decreases Thymus and Spleen Indices in CIA Rats

**Figure 4:**
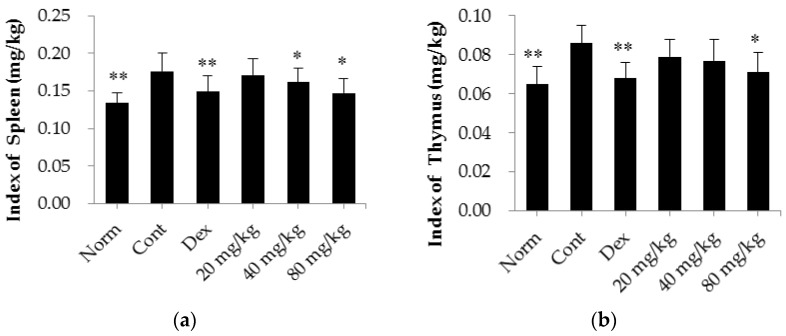
Effects of matrine on thymus and spleen indices in CIA rats. (**a**) Index of spleen; (**b**) Index of thymus. Norm: normal rats; Cont: control rats; Dex: rats treated with dexamethasone. The data are presented as the mean ± SD (*n* = 10); * *p* < 0.05, ** *p* < 0.01, compared with the Cont.

As shown in Figure 4, the thymus and spleen indices were significantly higher in CIA rats (*p* < 0.01) than in normal rats. After treatment with dexamethasone (0.05 mg/kg/d), both the thymus and the spleen indices were reduced (*p* < 0.01) compared with those of the control group. Similarly, treatment with matrine also reversed the increase in the thymus and spleen indices when administered at a dose of 80 mg/kg (*p* < 0.05, compared with the control group). In addition, when administered at a dose of 40 mg/kg, matrine significantly decreased the spleen index (*p* < 0.05, compared with the control group).

### 2.3. Matrine Decreases Inflammation as well as Joint and Synovial Tissue Destruction in CIA Rats

**Figure 5:**
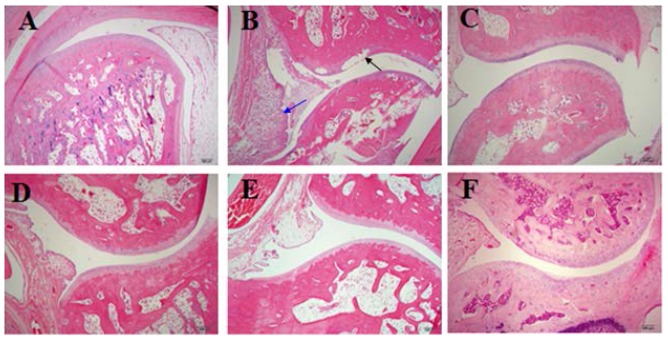
The effects of matrine on the histopathological characteristics of joints and synovial tissues in CIA rats (×40). **A**–**F**: normal rats (**A**); control RA rats (**B**), arrows indicate cell infiltration (blue) or bone erosion (black); RA rats treated with dexamethasone (**C**); RA rats treated with matrine at 20 (**D**); 40 (**E**); and 80 (**F**) mg/kg/days.

The results of histopathological examinations of the CIA rat joints and synovial tissues are shown in Figure 5. Intact articular cartilage and normal joints and synovial tissues were observed in the normal rats. However, severe bone destruction and inflammation were observed in the H&E sections of the control CIA rats. The results of the histological analyses indicated that inflammation as well as joint and synovial tissue destruction were significantly reduced after treatment with dexamethasone and matrine.

### 2.4. Matrine Decreases the Release of TNF-α, IL-6, IL-1β, IL-8, IL-17A and IL-10 into the Serum of CIA Rats

**Figure 6:**
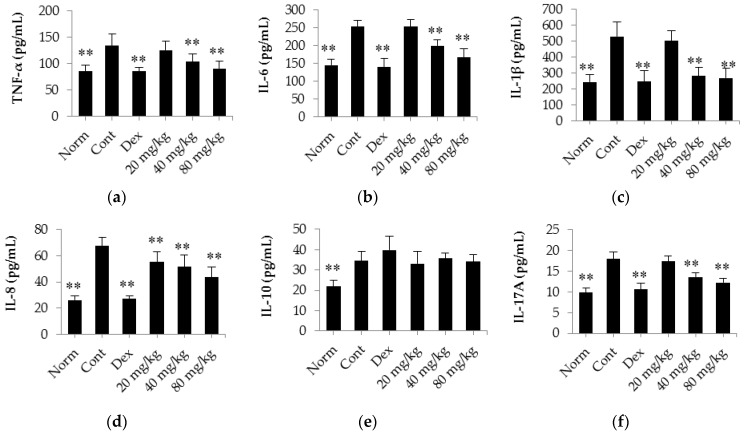
The effects of matrine on the release of TNF-α, IL-6, IL-1β, IL-8, IL-17A and IL-10 into the serum of CIA rats. (**a**) TNF-α content; (**b**) IL-6 content; (**c**) IL-1β content; (**d**) IL-8 content; (**e**) IL-10 content; (**f**) IL-17A content. Norm: normal rats; Cont: control rats; Dex: rats treated with dexamethasone. The data are presented as the mean ± SD (*n* = 10); ** *p* < 0.01, compared with the Cont.

The results shown in Figure 6 indicate that there was a clear increase in the levels of TNF-α, IL-6, IL-1β, IL-8, IL-17A and IL-10 in CIA rats (*p* < 0.01) compared with normal rats. However, the release of these regulatory cytokine (except for IL-10) was significantly decreased in rats treated with dexamethasone (0.05 mg/kg/days) compared with control CIA rats. Interestingly, daily treatment of CIA rats with matrine at a dose of 40 or 80 mg/kg significantly decreased the release of TNF-α, IL-6, IL-1β, IL-8, and IL-17A into the serum (*p* < 0.01) compared with the release observed in the control CIA rats. However, after treating the rats with matrine, no obvious effect on the release of IL-10 into the serum of CIA rats was observed (*p* > 0.05).

### 2.5. Matrine Decreases the Serum Levels of MMP-2, MMP-3 and MMP-9 in CIA Rats

**Figure 7:**
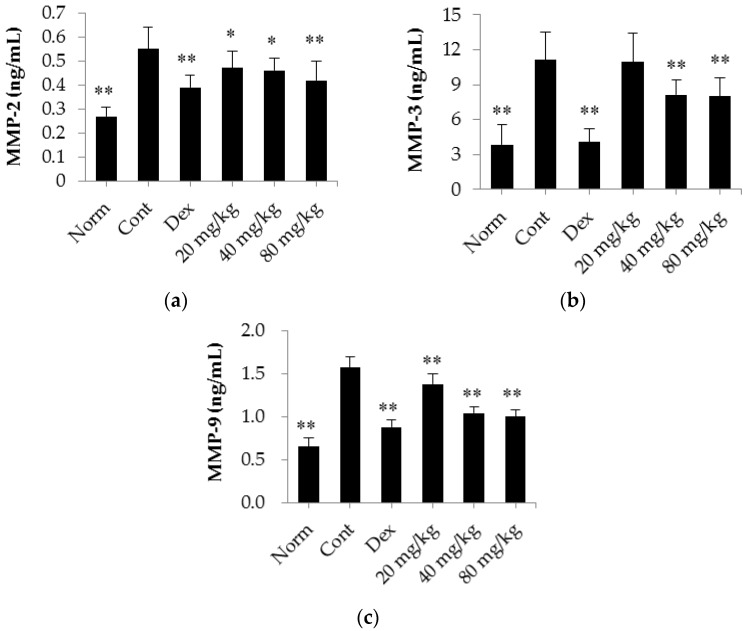
The effects of matrine on the expression of MMP-2, MMP-3 and MMP-9 in the serum of CIA rats. (**a**) MMP-2 content; (**b**) MMP-3 content; (**c**) MMP-9 content. Norm: normal rats; Cont: control rats; Dex: rats treated with dexamethasone. The data are presented as the mean ± SD (*n* = 10); * *p* < 0.05, ** *p* < 0.01, compared with the Cont.

Next, we analyzed the production of MMP-2, MMP-3 and MMP-9 in the serum of CIA rats. The results are shown in Figure 7. We found that the serum levels of MMP-2, MMP-3 and MMP-9 in the CIA rats were markedly elevated (*p* < 0.01) compared with those in the normal rats. In addition, we found that daily treatment with dexamethasone (0.05 mg/kg/d) significantly decreased MMP expression levels (*p* < 0.01 for MMP-2, MMP-3, and MMP-9) compared with those observed in the control CIA rats. Similarly, matrine also decreased the expression levels of MMP-2 and MMP-9 when administered at a dose of 20, 40 or 80 mg/kg/days (*p* < 0.05) compared with the expression levels observed in the control CIA rats. Moreover, MMP-3 expression was decreased in rats administered matrine at a dose of 40 or 80 mg/kg/d (*p* < 0.01) compared with control CIA rats.

### 2.6. Matrine Down-Regulates the Expression Levels of p-IκB, Cox-2, and iNOS and Up-Regulates the Expression Level of IκB in the Synovial Tissues of CIA Rats

**Figure 8:**
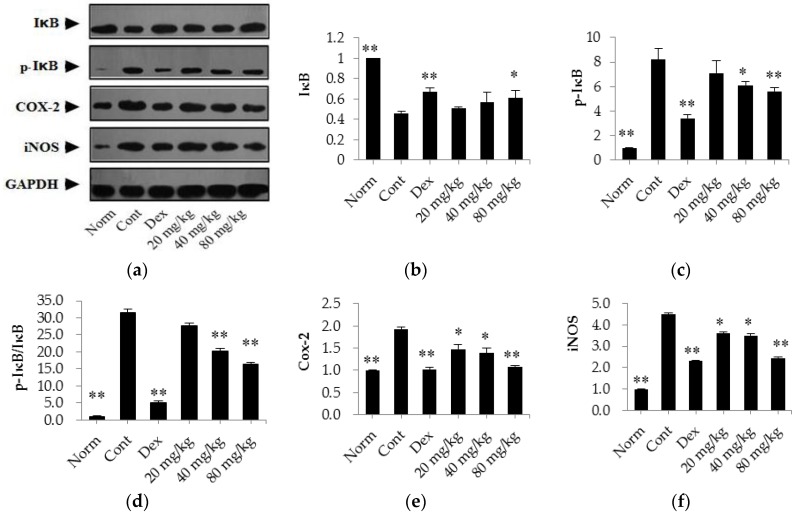
The effects of matrine on the expression of IκB, p-IκB, Cox-2, and iNOS in the synovial tissues of CIA rats. (**a**) Representative blots of IκB, p-IκB, Cox-2, and iNOS proteins; (**b**–**f**) Relative expression levels of IκB, p-IκB, IκB/p-IκB ration, Cox-2, and iNOS. Norm: normal rats; Cont: control rats; Dex: rats treated with dexamethasone. The data are presented as the mean ± SD (*n* = 4); * *p* < 0.05, ** *p* < 0.01, compared with the Cont.

In the present investigation, we also evaluated the protein expression levels of IκB, p-IκB, Cox-2, and iNOS in synovial tissues of CIA rats using western blot assays. As shown in Figure 8, our results indicate that the expression level of IκB was significantly lower in control CIA rats (*p* < 0.01) than in normal rats. However, the expression levels of p-IκB, Cox-2, and iNOS were markedly up-regulated (*p* < 0.01) in these rats compared with those levels in normal rats. After treatment with dexamethasone (0.05 mg/kg/days), the expression levels of p-IκB, Cox-2, and iNOS were significantly down-regulated (*p* < 0.01) and the expression of IκB was elevated (*p* < 0.01) compared with levels in the control CIA rats. Furthermore, we also found that matrine (80 mg/kg) decreased the expression levels of p-IκB, Cox-2, and iNOS and increased the expression of IκB (*p* < 0.01) compared with the levels observed in the control CIA rats. Additionally, when administered at a dose of 40 mg/kg, matrine decreased the expression of p-IκB, Cox-2, and iNOS (*p* < 0.05) but had no effect on the expression of IκB (*p* > 0.05). Moreover, when administered at a dose of 20 mg/kg, matrine decreased the expression of Cox-2 and iNOS (*p* < 0.05) but did not affect the expression of IκB and p-IκB (*p* > 0.05) compared with the levels in the control CIA rats.

## 3. Discussion

An increasing number of investigations indicate that naturally derived agents might be useful as novel candidate drugs for treating RA [10,18]. To the best of our knowledge, the present investigation is the first report to evaluate the therapeutic effects of matrine on RA in the CIA animal model. Our results demonstrate that matrine possesses promising anti-arthritic properties that affect type II collagen-induced arthritis in rats by inhibiting inflammatory responses.

Currently, it is generally recognized that rheumatoid arthritis (RA) is an immune-mediated disease with chronic progressive inflammation [19,20]. In addition, RA animal models have been extensively investigated, and the CIA and adjuvant-induced arthritis (AIA) models are the two that are most commonly used to study RA [21]. Furthermore, CIA is a well-described RA animal model that induces immunological and pathological features similar to those in the RA disease observed in humans [21,22,23]. In the present study, we successfully established a CII-induced arthritis rat model, and we used these CIA rats to evaluate the anti-arthritic activity of matrine. Our results demonstrate that treatment with matrine decreased paw swelling, arthritis indices and weight loss in CIA rats. In addition, daily treatment of CIA rats with matrine for 30 days alleviated inflammation and the destruction of joints and synovial tissues and decreased the indices of the thymus and spleen. These results suggest that matrine may have potential therapeutic effects on CIA.

While inflammatory reactions are not the only features of RA, they are the main problem in RA patients. Therefore, controlling inflammatory reactions might be a feasible RA treatment method [18,21,24]. Our results demonstrate that matrine decreased inflammatory responses during the development of RA, including alleviating paw swelling, decreasing arthritis indices, and reducing inflammation in the joints and synovial tissues. To explore the pharmacological mechanism of matrine, we investigated the effect of marine on the release of pro-inflammatory cytokines and the expression of inflammatory response-related proteins. Pro-inflammatory cytokines, such as TNF-α, IL-1β, IL-6, IL-8 and IL-17A, stimulate inflammatory responses in arthritic joints and synovial tissues; as such, these cytokines have been reported as potential therapeutic targets for RA [3,25,26,27,28]. The present study shows that daily treatment with matrine for 30 days suppressed the levels of these pro-inflammatory cytokines in the serum of CIA rats. In addition, these pro-inflammatory cytokines have also been reported to promote the secretion of MMPs [10,18,29]. MMPs belong to the zinc- and calcium-dependent endopeptidase family, and they are known to be produced by synovial fibroblasts. Increased MMP levels can result in the digestion of fibrillar collagens and the degradation of extracellular matrix substrates, leading to damage in joints and synovial tissues [18,30]. Our research shows that treatment with matrine decreased the expression of MMP-2, MMP-3 and MMP-9 in the serum of CIA rats. Therefore, in combination with the results of histopathological examinations of joints and synovial tissues, these results suggest that matrine exerts a strong protective effect in the joints and synovial tissues of CIA rats.

Pro-inflammatory enzymes, including Cox-2 and iNOS, have also been reported to be important for the development of inflammatory responses, and they are over-expressed during the inflammatory process. In addition, these enzymes cause the synthesis and secretion of prostaglandins, including PGE2, which is closely related to pain and inflammatory symptoms and can lead to pain and inflammation in the joints of RA patients [10]. In this investigation, we found that matrine inhibited the release of both Cox-2 and iNOS. These results provide further support for the therapeutic effects of matrine. The NF-κB pathway is a classical pathway involved in the development and regulation of inflammatory reactions, and NF-κB has also been reported to be up-regulated in the joints and synovial tissues of RA patients [18,30,31]. NF-κB proteins are normally sequestered in the cytosol as inactive complexes, where they are bound by IκB-α, an NF-κB-inhibiting factor. In previous studies, IκB-α was inactivated by ubiquitin-mediated degradation following the activation of inflammatory response signals, and this inactivation resulted in the release of NF-κB. Upon activation, NF-κB translocated to the nucleus to act as a transcription factor [32,33]. The western blot results in the current study showed that daily treatment with matrine for 30 days down-regulated the expression of Cox-2 and iNOS and up-regulated the expression of IκB. These results indicate that matrine treatment down-regulates the expression of Cox-2 and iNOS, which might be related to its inhibitory effect on NF-κB pathway activation.

## 4. Materials and Methods

### 4.1. Plant Material

*Sophora flavescens* roots were obtained from the Anguo Market of Traditional Chinese Herbs (Hebei, China) and authenticated by Lu-Ping Qin at the Second Military Medical University (Shanghai, China). The voucher specimen of this plant is kept at the Herbarium of the Department of Pharmacognosy, School of Pharmacy, Second Military Medical University, Shanghai, China (No. SF20140907).

### 4.2. Animals

Male Sprage Dawley (SD) rats (170 ± 10 g) were purchased from the Experimental Animal Center of the Second Military Medical University (Shanghai, China). They were housed at 21 ± 1 °C in a facility under a 12-h light/dark cycle with free access to a standard pellet diet and tap water. All animal experiments in our present research were performed in accordance with the National Institutes of Health Guide for the Care and Use of Laboratory Animals, and our experimental protocols were approved by the Animal Care and Use Committee of the Second Military Medical University (2015LY014, 4 January 2015).

### 4.3. Chemicals and Reagents

Chicken type II collagen (CII) was purchased from Xi’an Herb King Biotechnology Co., Ltd. (Xi’an, China); dexamethasone, complete Freund’s adjuvant (CFA) and incomplete Freund’s adjuvant (IFA) were purchased from Sigma-Aldrich Co. (Shanghai, China); rat matrix metalloproteinase (MMP)-9 (cat. No. ERC018.48) ELISA kit was purchased from NeoBioscience Technology Co., Ltd. (Beijing, China); rat interleukin (IL)-10 (cat. No. CSB-E04595r), rat tumor necrosis factor (TNF)-α (cat. No. CSB-E11987r), rat IL-8 (cat. No. CSB-E07273r) and rat MMP-3 (cat. No. CSB-E07410r) ELISA kits were purchased from CUSABIO Biotech Co. (Wuhan, China); rat IL-1β (cat. No. ab100768), rat IL-6 (cat. No. ab100772) and rat IL-17A (cat. No. ab119536) ELISA kits were purchased from the Abcam Co. (Cambridge, UK); rat MMP-2 ELISA kits (cat. No. EA100413) was purchased from OriGene Technologies, Inc. (Rockville, MD, USA); inhibitor of κB (IκB)-β (Phospho-Ser23) antibodies, IκB-β (Ab-23) antibodies, cyclooxygenase (COX)-2 rabbit polyclonal antibodies, inducible nitric oxide synthase (iNOS) (Ab-151) antibodies, GAPDH antibodies and horseradish peroxidase (HRP)-conjugated goat anti-rabbit IgG secondary antibodies were purchased from EnoGene Biotech (Shanghai, China). BCA protein assay reagents were purchased from Beyotime Co. (Haimen, China). All other chemicals used in this study were of analytical reagent grade.

### 4.4. Preparation of Matrine

**Figure 9:**
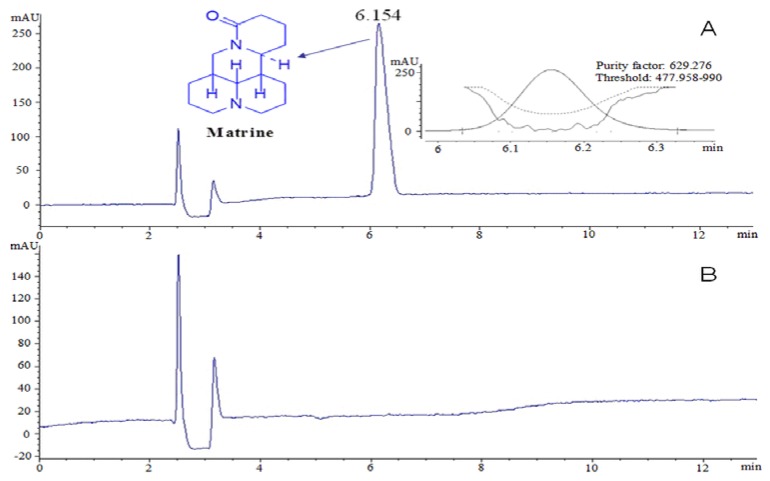
The HPLC profile of matrine. (**A**) sample; (**B**) solvent.

Dried *S. flavescens* roots were powdered and extracted three times under reflux using 70% aqueous ethanol (each extraction period lasted 1.5 h). The solvent was evaporated under vacuum to yield crude total extracts. Then, the extracts were dissolved in 2% HCl (pH 3.5) and partitioned with ether. The pH of the aqueous solution was re-adjusted with NH4OH to 9.0, and the solution was then extracted with CHCl3. The CHCl3 extract (crude alkaloid) was subjected to silica gel (100–200 mesh) column chromatography and eluted using ethyl acetate-methanol (20:1~1:2) to obtain six fractions, referred to as fractions A–F. Using a series of chromatographic techniques, such as silica gel column chromatography (200–300 mesh) and Sephadex LH-20 chromatography, matrine was isolated from fraction C and identified using NMR (Figure S1). In addition, the purity of the matrine obtained in our study was over 90%, according to the HPLC area normalization method; this result was further confirmed by our findings that the purity factors were within the scope of the threshold value and that the threshold curves did not intersect the purity curves [34] (Figure 9).

### 4.5. CII-Induced Arthritis (CIA) Animal Model Preparation and Experiment Protocols

To evaluate the potential anti-arthritic effects of matrine, a total of sixty rats were randomly divided into the following six groups (*n* = 10): A. normal: normal rats treated with saline (10 mL/kg); B. control: CIA rats treated with saline (10 mL/kg); C. dexamethasone: CIA rats treated with dexamethasone (positive drug, 0.05 mg/kg); D. matrine (20 mg/kg): CIA rats treated with matrine at a dose of 20 mg/kg; F. matrine (40 mg/kg): CIA rats treated with matrine at a dose of 40 mg/kg; and E. matrine (80 mg/kg): CIA rats treated with matrine at a dose of 80 mg/kg.

The CIA rat model was prepared as previously described [35], with minor modifications. In brief, CII was dissolved in 0.1 mM acetic acid to achieve a final concentration of 4 mg/mL. Then, the CII solution was emulsified with an equal volume of CFA. Rats were initially immunized by a subcutaneous injection of the CII emulsion into the tail root (100 μL/rat). After two weeks, the rats were secondarily immunized by subcutaneously injecting the CII emulsion with an equal volume of IFA at the same location (100 μL/rat). After approximately 20 days, the immunized rats showed clear symptoms of RA at their toe joints, including inflammation, erythema and swelling.

At 21 days after the initial immunization with CII, the rats were orally treated with saline, dexamethasone or different doses of matrine. During the experiment, the body weights and paw volumes of the rats were measured every 3 days. In addition, the arthritis indices of the rats were measured every three days using the following ordinal scale: (0) unaffected; (1) 1 type of joint affected; (2) 2 types of joints affected; (3) 3 types of joints affected; and (4) 3 types of joints affected plus maximal erythema and swelling [10]. After the treatments were administered for 30 days, blood samples were collected using an orbital blood sampling protocol, and the rats were then sacrificed via decapitation. The thymus and spleen were dissected from each mouse to determine their visceral indices, i.e., the ratio (mg/g) of thymus or spleen wet weight to body weight. In addition, synovial tissues were collected for western blot assays, and hind paws and knee joints were collected for histopathological examination.

### 4.6. Determination of Serum Cytokine Levels

Serum samples were prepared after they were incubated under ice-cold conditions and then centrifuged for 15 min (1800× *g*). The samples were stored at −80 °C until analysis. Then, the serum levels of TNF-α, IL-1β, IL-6, IL-8, IL-10, IL-17A, MMP-2, MMP-3 and MMP-9 were determined using commercial ELISA kits according to the manufacturer’s protocols and instructions.

### 4.7. Histopathological Examination

For histopathological evaluation, tissue joint specimens were stained with hematoxylin and eosin (H & E). Hind paws and knee joints were fixed in 10% formaldehyde and were then embedded in paraffin. Subsequently, 3-μm-thick tissue sections were cut, de-paraffinized and rehydrated using standard techniques. Finally, the tissue sections were stained with H & E [36]. Histopathological analyses were performed using microscopy (Olympus, Tokyo, Japan).

### 4.8. Western Blot Assays

After the synovial tissue samples were homogenized, total protein was extracted from the homogenates. Protein concentrations were determined using a BCA protein assay. Equal amounts of protein (30 μg) from each sample were loaded and run on 10% sodium dodecyl sulfate-polyacrylamide electrophoresis gels (SDS-PAGE) and then transferred to polyvinylidene difluoride (PVDF) membranes. The membranes were incubated with p-IκB, IκB, COX-2, iNOS and GAPDH primary antibodies, followed by HRP-conjugated goat anti-rabbit IgG secondary antibodies, to detect the corresponding proteins. Finally, immunoreactive bands were visualized using an ECL detection reagent (Bio-Rad, Hercules, CA, USA). To normalize protein loading, protein expression levels were expressed relative to the level of GAPDH. The protein bands densitometry were determined by using a ChemiDoc XRS gel imaging system (Bio-Rad) and analyzed by Quantity One software (Bio-Rad).

### 4.9. Statistical Analysis

The data were evaluated using one-way ANOVA followed by Dunnett’s multiple comparisons tests (between different groups) and are presented as the mean ± S.D. The statistical significance of observed differences was determined using SPSS software (SPSS for Windows 18.0, SPSS Inc., Chicago, IL, USA). Differences were considered to be significant at *p* < 0.05.

## 5. Conclusions

In conclusion, the present investigation demonstrates that matrine exerts strong anti-arthritic effects on type II collagen-induced arthritis in rats. The potential pharmacological mechanism for these effects might be related to the inhibition of inflammatory reactions during the RA process, whereby matrine suppresses the release of pro-inflammatory cytokines and enzymes and inhibits the promotion of proteins in the NF-κB pathway. Therefore, our results suggest that matrine may be a novel candidate drug for clinically treating RA.

## Supplementary Materials

Supplementary materials can be found at www.mdpi.com/1422-0067/17/9/1410/s1.
