# Supplementary material for: Matrine Exerts a Strong Anti-Arthritic Effect on Type II Collagen-Induced Arthritis in Rats by Inhibiting Inflammatory Responses
Source: Int J Mol Sci. 2016 Aug 26;17(9):1410. doi: 10.3390/ijms17091410 (PMC5037690; doi:10.3390/ijms17091410)
Supplement: Supplementary file 1 [file ijms-17-01410-s001.pdf]

Supplementary Materials: Matrine Exerts a Strong Anti-Arthritic Effect on Type II Collagen-Induced Arthritis in Rats by Inhibiting Inflammatory Responses

Jiang Pu, Fan-Fu Fang, Xiu-Qing Li, Zhi-Heng Shu, Yi-Ping Jiang, Ting Han, Wei Peng and Cheng-Jian Zheng

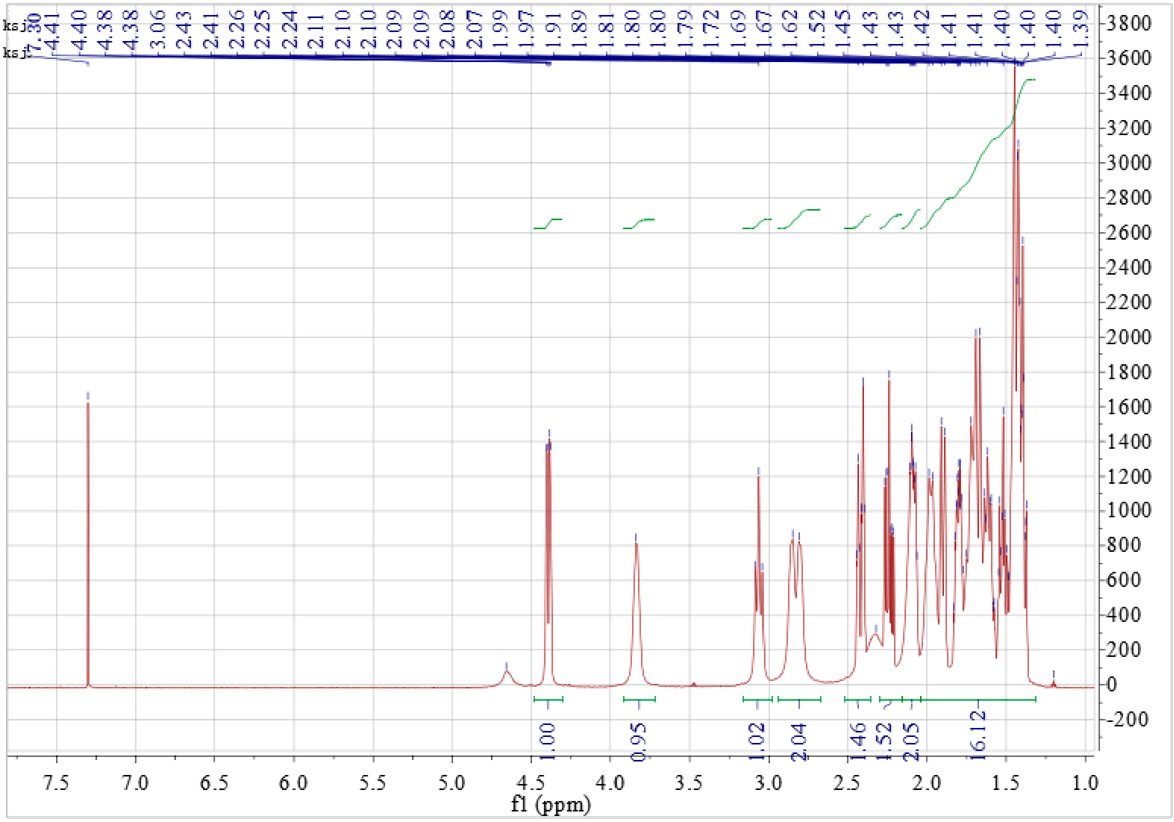

(**a**)

Figure S1. *Cont.*

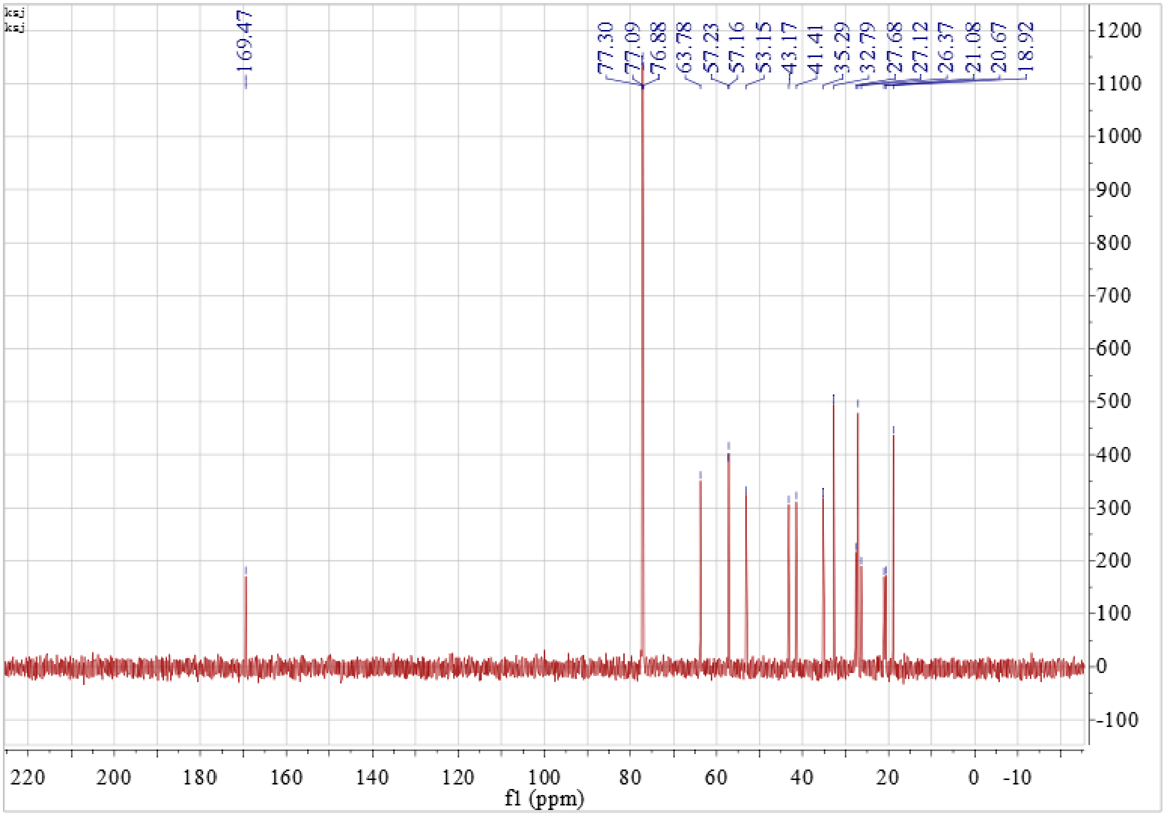

(**b**)

Figure S1. ^{1}H-NMR (**a**) and ^{13}C-NMR (**b**) spectrum of matrine.

1. Materials and Methods

1.1. HPLC Analysis of Matrine

Matrine was dissolved in methanol prior to HPLC analysis. This was performed on an Agilent-1100 system with a Zorbax Extend-C18 chromatographic column (250 mm × 4.6 mm, 5 μm) at 30 °C with a H_2O (+0.2% triethylamine and 0.1% phosphoric acid) (A)/acetonitrile (B) elution system, a sample injection volume of 20 μL, flow rate of 1.0 mL/min, and a detection wavelength 220 nm. Samples were analyzed by using a fixed gradient program as follows: 3% B and 97% A within 13 min.

1.2. NMR Measurement

Matrine was dissolved in $CDCl_3$ prior to NMR analysis. NMR spectra were recorded on a Bruker Avance 600 NMR spectrometer with TMS as an internal standard.
